# Supplementary material for: Superlinear scaling of riverine biogeochemical function with watershed size
Source: Nat Commun. 2022 Mar 9;13:1230. doi: 10.1038/s41467-022-28630-z (PMC8907334; doi:10.1038/s41467-022-28630-z)
Supplement: Supplementary file 1 — Supplemental Information [file 41467_2022_28630_MOESM1_ESM.pdf]

Supplementary Material for "Superlinear scaling of riverine biogeochemical function with watershed size"

Supplementary Notes

Note 1. Length and Stream Surface Area Scaling With Increasing Watershed Area

The scaling of length and stream surface area (SA) in the three theoretical channel networks (i.e., square, rectangular, and narrow, Supplementary Table 1) were similar to those in real networks. Cumulative length scaled nearly linearly with increasing watershed area in the three differently shaped river networks (length slope = 0.97, 1.0, 1.05 in square, rectangular, and narrow network structures/shapes, respectively) and seven real river networks (mean length slope = 1.06, range 0.99-1.16 [1]; Supplementary Figure 1A). In contrast, cumulative SA scaled superlinearly with increasing watershed size using the parameters from the theoretical channel networks (SA slope = 1.11-1.23, 1.14-1.27, 1.25-1.41 in square, rectangular, and narrow, respectively, with f in Equation 3 ranging from 0.4 to 0.6) and real river networks (mean SA slope = 1.23, range 1.1 – 1.4, based on measured width vs. discharge relationships in [1]; Supplementary Figure 1B). Superlinearity is enhanced if the width versus discharge slope (f in $w = eQ^f$) is higher. For example, in the theoretical rectangular network, the slope for scaling SA increases from 1.14 to 1.27 as f increases from 0.4 to 0.6 (typical range [2]). Superlinearity of SA occurs in all watersheds, but is enhanced in narrower networks because small streams are more likely to enter directly into larger streams, and in watersheds with greater increases in width as flow increases in the downstream direction.

Note 2. Effect of biogeochemical reaction rate magnitude on superlinearity across flow conditions

Superlinear scaling occurs over a broader range of flows (material supply) for processes with lower υ_f (*b* in Equation 2; Supplementary Figure 6, Fig. 5). Under higher reaction rates, shunting of material downstream requires higher flows, and implies a greater degree of source limitation in larger downstream ecosystems. Thus, the scaling of riverine biogeochemical function with increasing watershed size will depend on demand for the constituent of interest, stoichiometric and energetic constraints on function, as well as flow conditions (the balance of material supply and demand). Processes with relatively slow reaction rates like bulk DOC mineralization or denitrification (typical values ~35 m yr^{-1}) [11,12] are more likely to scale superlinearly compared to those with faster rates such as NH_4^+ uptake (Supplementary Figure 6, Fig. 5).

Note 3. Effect of local biogeochemical reaction rate trending with increasing river size

Although there is little empirical evidence that first-order reaction rates, such as nutrient uptake, change systematically along the river continuum [3,4], theoretical considerations [5,6], models [7,8], and some measurement syntheses [9] (Table S5) suggest that reaction rates could be a function of stream size (*m.local* $\neq 0$), similar to observations of GPP and ER [10]. Varying *m.local* (from -0.1 to 0.1; $\upsilon_f = \upsilon_{f.1km2} A^{m.local}$, where $\upsilon_{f.1km2}$ is the normalization constant in Equation 2) did not affect the scaling of cumulative function with increasing watershed area at low flows (supply << potential demand; mean $d = 0.97$; Supplementary Figure 5). However, at high flow, cumulative processing becomes increasingly superlinear (mean *d* increases from 1.11 to 1.22) as *m.local* increases from -0.1 to 0.1. Thus, trends in process rates with increasing stream size influence the

scaling relationship at higher flow conditions when greater fluxes occur, due to the distribution of network-scale demand relative to material supply.

Note 4. Scaling of river network function with ecosystem size rather than watershed size

The allometric scaling model applies to riverine biogeochemical function with watershed size rather than ecosystem size (i.e., defined by riverine surface area) because of the relevance of the former for understanding the role of river networks in the Earth system. Previous analyses of other aquatic ecosystems like estuaries and lakes [13,14] emphasized scaling with ecosystem size defined by the volume of the water body (V) or benthic surface area (SA). For example, Nidzieko [14] found that in deeper estuaries, cumulative gross primary production (P) ~ $V^{0.75}$ (similar to organisms) and P ~ $SA^{0.91}$, while in shallower estuaries, P ~ $V^{0.90}$ and P ~ $SA^{0.94}$. These sublinear scaling relationships were attributed to transport limitation of required nutrients. Theoretical work on fractal transport networks predicts that the transport capability of the network should scale linearly with SA for benthic systems and sublinearly with V for pelagic systems, consistent with these ecosystem observations.

Though water-column processes can become important in some very large rivers, biogeochemical activity across most river sizes occurs in the benthos [15], so comparison with the benthic area scaling described in [14] is most relevant. When concentration is not a factor (i.e., zero-order kinetics), scaling of cumulative riverine function with SA is merely a function of how SA and local process rates (*m.local*) scale (Equation 2, Fig. 1). Thus, the size scaling relationship in the zero-order scenario is not directly comparable to the allometric scaling in other ecosystems. When first-order kinetics apply, our results demonstrate similar sublinear scaling of cumulative riverine function with increasing SA (as opposed to increasing watershed area

emphasized in the main text) during low flow (low supply) conditions. The scaling of cumulative function vs. accumulating SA approaches linearity under high flow (high supply) conditions. For example, across all scenarios analyzed for a rectangular river network (Supplementary Table 3), the mean slope of cumulative riverine function vs. cumulative SA (not watershed area as in the main text) increases from 0.85 to 0.98 as watershed runoff increases from 0.1 to 20 times the mean annual runoff, encompassing the values identified by [14] in estuaries for equilibrium conditions. At low flows, river network processes in the larger downstream portion of the network are supply-limited because concentration (C) declines moving downstream (recall the reaction rate parameter $\upsilon_f = U/C$). At high flows, the SA over the entire river network operates at its maximum because uptake processes in upstream portions of the network minimally reduce concentrations in the downstream direction. During high flows, surface water concentrations throughout the entire network are relatively unaltered from their input concentrations, and the network as a whole has minimal effect on export fluxes. Although the scaling exponent in our analyses is similar to previous work in individual ecosystems (e.g., linear scaling when source limitation is minimized during high flows), we emphasize that the scaling relationships we identified for river networks are derived based on a different set of assumptions about inputs (mostly to headwaters), transport (upstream to downstream, with processes en route), and biological activity that are consistent with riverine systems. Finally, we emphasize that the model used here represents river channels only and does not encompass other freshwater features within river networks (ponds, lakes, reservoirs, floodplains). These other components of aquatic networks must be considered to fully understand how scaling varies across flow conditions.

Note 5. Comparison to Allometric Scaling in the Metabolic Theory of Ecology

River networks are in some ways analogous to the fractal networks of organisms described by the metabolic theory of ecology (MTE) [16,17]. However, transport processes in rivers differ fundamentally from organisms in ways that influence scaling. First, the MTE assumes that biological transport networks optimize efficiency of material transport to maximize metabolism. In contrast, river network structure optimizes water and sediment transport [18], while riverine biological communities develop longitudinally within this context to maximize energy capture along the upstream-downstream continuum [6]. Second, the MTE assumes that a network's primary function is to transport, rather than transform materials. River networks clearly differ in this respect, as substantial processing can occur during transport [11,19–21]. Third, observed sublinear scaling in organisms is due to the three-dimensional, space-filling attribute of transport networks. In contrast, river networks are fractal in two dimensions. Fourth, whereas organismal transport networks are *distributary*, moving resources in concentrated forms from intake to cells, river networks *aggregate* resources from diffuse inputs throughout the full network. As a result, scaling relationships for river networks are likely to differ from those described by the MTE. We note that this study did not explicitly consider the role of temperature, the other major component emphasized in the MTE, but because reaction rates are often highly temperature-dependent, following a $Q_{10} = 2$ or Boltzmann's relationship, this effect could be explored by varying reaction rates using the scaling framework presented here.

Supplementary References

1. Rüegg, J. *et al.* Baseflow physical characteristics differ at multiple spatial scales in stream networks across diverse biomes. *Landsc. Ecol.* **31**, 119–136 (2016).
2. Knighton, D. Fluvial forms and processes: a new perspective. (1998).
3. Ensign, S. H. & Doyle, M. W. Nutrient spiraling in streams and river networks. *J. Geophys. Res.* **111**, G04009 (2006).
4. Tank, J. L., Rosi-Marshall, E. J., Baker, M. A. & Hall, R. O. Are rivers just big streams? A pulse method to quantify nitrogen demand in a large river. *Ecology* **89**, 2935–2945 (2008).
5. Raymond, P. A. *et al.* Scaling the gas transfer velocity and hydraulic geometry in streams and small rivers. *Limnol. Oceanogr. Fluids Environ.* **2**, 41–1597669 (2012).
6. Vannote G.W. Minshall, K.W. Cummins, J.R. Sedell and C.E. Cushing, R. L. The river continuum concept. *Can. J. Fish. Aquat. Sci.* **37**, 130–137 (1980).
7. Alexander, R. B., Smith, R. A. & Schwarz, G. E. Effect of stream channel size on the delivery of nitrogen to the Gulf of Mexico. *Nature* **403**, 758–761 (2000).
8. Moore, R. B., Johnston, C. M., Smith, R. A. & Milstead, B. Source and delivery of nutrients to receiving waters in the northeastern and mid-atlantic regions of the United States. *J. Am. Water Resour. Assoc.* **47**, 965–990 (2011).
9. Hall, R. O., Baker, M. A., Rosi-Marshall, E. J., Tank, J. L. & Newbold, J. D. Solute-specific scaling of inorganic nitrogen and phosphorus uptake in streams. *Biogeosciences* **10**, 7323–7331 (2013).
10. Finlay, J. C. Stream size and human influences on ecosystem production in river networks. *Ecosphere* **2**, art87 (2011).

11. Mulholland, P. J. *et al.* Stream denitrification across biomes and its response to anthropogenic nitrate loading. *Nature* **452**, 202–205 (2008).

12. Howarth, R. W. *et al.* Regional nitrogen budgets and riverine inputs of N and P for the drainages to the North Atlantic Ocean: natural and human influences. *Biogeochemistry* **35**, (1996).

13. Schramski, J. R., Dell, A. I., Grady, J. M., Sibly, R. M. & Brown, J. H. Metabolic theory predicts whole-ecosystem properties. *Proc. Natl. Acad. Sci. U. S. A.* **112**, 2617–22 (2015).

14. Nidzieko, N. J. Allometric scaling of estuarine ecosystem metabolism. *Proc. Natl. Acad. Sci. U. S. A.* **115**, 6733–6738 (2018).

15. Gardner, J. R., Pavelsky, T. M. & Doyle, M. W. The Abundance, Size, and Spacing of Lakes and Reservoirs Connected to River Networks. *Geophys. Res. Lett.* **46**, 2592–2601 (2019).

16. West, G. B., Brown, J. H. & Enquist, B. J. The Fourth Dimension of Life: Fractal Geometry and Allometric Scaling of Organisms. *Science (80-.).* **284**, 1677–1679 (1999).

17. Brown, J. H., Gillooly, J. F., Allen, P. A., Savage, V. M. & West, G. B. Toward a Metabolic Theory of Ecology. *Ecology* **85**, 1771–1789 (2004).

18. Rodríguez-Iturbe, I. *et al.* Energy dissipation, runoff production, and the three-dimensional structure of river basins. *Water Resour. Res.* **28**, 1095–1103 (1992).

19. Bernhardt, E. S., Likens, G. E., Buso, D. C. & Driscoll, C. T. In-stream uptake dampens effects of major forest disturbance on watershed nitrogen export. *Proc. Natl. Acad. Sci.* **100**, 10304–10308 (2003).

20. Peterson, B. J. *et al.* Control of nitrogen export from watersheds by headwater streams. *Science (80-.).* **292**, 86–90 (2001).

21. Hotchkiss, E. R. *et al.* Sources of and processes controlling CO 2 emissions change with the size of streams and rivers. *Nat. Geosci.* **8**, 696–699 (2015).

22. Wollheim, W. M., Peterson, B. J., Thomas, S. M., Hopkinson, C. H. & Vörösmarty, C. J. Dynamics of N removal over annual time periods in a suburban river network. *J. Geophys. Res.* **113**, G03038 (2008).

23. Samal, N. *et al.* Projections of Coupled Terrestrial and Aquatic Ecosystem Change Relevant to Ecosystem Service Valuation at Regional Scales. *Ecol. Soc.* **22**, 18. https://doi.org/10.5751/ES-09662–220418 (2017).

24. Ulseth, A. J. *et al.* Distinct air–water gas exchange regimes in low- and high-energy streams. *Nat. Geosci.* **12**, 259–263 (2019).

25. Mineau, M. M. *et al.* Dissolved organic carbon uptake in streams: A review and assessment of reach-scale measurements. *Limnol. Oceanogr.* **121**, (2016).

26. Georgian, T. *et al.* Comparison of corn pollen and natural fine particulate matter transport in streams: Can pollen be used as a seston surrogate? *J. North Am. Benthol. Soc.* **22**, 2–16 (2003).

27. Wollheim, W. M. *et al.* Influence of stream size on ammonium and suspended particulate nitrogen processing. *Limnol. Oceanogr.* **46**, 1–13 (2001).

28. Helton, A. M., Hall, R. O. & Bertuzzo, E. How network structure can affect nitrogen removal by streams. *Freshw. Biol.* **63**, 128–140 (2018).

Supplementary Tables

Supplementary Table 1. Parameters used to define scenarios of river network characteristics for scaling cumulative riverine biogeochemical function vs. watershed area. Maximum watershed area in each scenario is standardized at 6321 km^2. R_a, R_b, and R_l are the river network geomorphological (structure) parameters that define the drainage areas (R_a), numbers (R_b), and mean lengths (R_l) of each river order in a network as well as flow path probabilities. *e* and *f* define channel widths at mean annual discharge of each river order ($w = eQ^f$), while *g* defines how width changes from the mean annual width with change in flow locally (e.g. the local hydrograph). *m.local* defines how local biogeochemical process rates (on a per unit surface area basis) change with increasing river size defined by drainage area.

Variable	*Abbreviation*	*Value*	*Source*
Network Geomorphology	Square	R_a=4.7, R_b=4.1, R_l=2.2	
	Rectangular	R_a=5.1, R_b=4.6, R_l=2.5	Helton et al. (2017)
	Narrow	R_a=7.4, R_b=6.0, R_l=4.4	
Channel Hydraulics	*e*	4, 8, 12	
	f	0.4, 0.5, 0.6	Knighton (1998)
	g	0, 0.1, 0.2	
Local Biogeochemical Rate Change with River Size			
- *zero-order*	*m.local* (zero-order)	-0.5, 0, 0.5	Assumption, constrained by Finlay (2011)
- *first-order*	*m.local* (first-order)	-0.1, 0, 0.1	Assumption

Supplementary Table 2. Empirical river networks and their structural and hydraulic attributes estimated from digital elevation models and local measurements. Attributes were used to estimate length and surface area scaling with watershed area in Supplementary Figure 1. A_1 is the mean area at the outlet of a first order watershed. L_1 is the mean length of a first order stream. R_A is the area ratio. R_B is the numbers ratio. R_L is the length ratio. f is the width vs. discharge power slope in the downstream direction. All networks are from [1], except the Ipswich R. network [22] and the Merrimack R. network [23].

River Network	***Biome***	***Watershed Area (km²)***	***A_1 (km²)***	***L_1 (km)***	***R_A***	***R_L***	***R_B***	***f***
Oksrukuyik Cr. (AK, USA)	Tundra	72.4	0.93	1.0	4.2	1.5	3.8	0.51
Caribou Cr. (AK, USA)	Boreal	103.6	1.5	1.1	4.4	2.7	3.3	0.51
Kings Cr. (KS, USA)	Prairie	16.3	0.15	0.40	5.0	2.0	4.0	0.26
Coweeta Cr. (NC, USA)	Temperate	15.7	0.03	0.18	6.2	2.6	4.3	0.36
R. Mameyes (PR, USA)	Tropical	22.6	0.13	0.38	3.5	1.7	3.3	0.4
Ipswich R. (MA, USA)	Temperate	404	0.52	0.65	4.9	2.7	4.5	0.5
Merrimack R. (NH, USA)	Temperate	13,031	1.7	1.6	4.4	2.1	4.6	0.58

Supplementary Table 3. Example of rectangular river network structure assuming $R_a = 5.1$, $R_b = 4.6$ $R_l = 2.5$, $A_1 = 1\ km^2$, and $L_1 = 1$ km, in a watershed area = 6321 km^2. For this watershed size and set of network structural parameters, only a partial 7th order network occurs, which is reflected in the scaled down 7th order length and number of 6th order tributaries. Direct inputs represent the proportion of total watershed inputs from the landscape where they first enter each stream order.

Stream Order	***Mean Length (km)***	***Mean Area (km2)***	***Numbers***	***Direct Inputs (proportion)***
1	1.0	1.0	3403.4	0.54
2	2.5	5.1	739.9	0.22
3	6.3	26.0	160.8	0.12
4	15.6	132.7	35.0	0.06
5	39.1	676.5	7.6	0.03
6	97.7	3450.3	1.7	0.02
7	87.7	6321.0	1.0	0.01

Supplementary Table 4. Example of flow path probabilities for the rectangular river network shown in Supplementary Table 3. Each row adds up to 1, accounting for the all the path's taken by streams of a given order.

Receiving Stream Order

Source Stream Order		**1**	**2**	**3**	**4**	**5**	**6**	**7**
	**1**	-	0.718	0.142	0.071	0.037	0.023	0.009
	**2**		-	0.718	0.143	0.074	0.046	0.018
	**3**			-	0.721	0.149	0.093	0.037
	**4**				-	0.737	0.188	0.074
	**5**					-	0.840	0.160
	**6**						-	1
	**7**							-

Supplementary Table 5. Synthesis of biogeochemical process rates as uptake velocity (υ_f) and local scaling with river size based on discharge at the time of measurement. Regression parameters describe the log-log relationship between uptake velocity and discharge ($\upsilon_f = bQ^{m.local}$). Q is used to approximate for A in the regressions. DOC = dissolved organic carbon. FPOM = fine particulate organic matter. All observed rates are shown in Supplementary Figure 5.

Constituent	*n*	*Median (m/yr)*	*Interquartile Range*	*b (log-log)*	*m.local (log-log)*	*p*	*R^2*	*Sources*
Gas exchange - all	711	1536	729-4020	3.27	-0.08	0.003	0.01	[24a]
Gas exchange - mountain	54	51082	23102-183960	5.25	0.70	2E-16	0.76	[24b]
NO_3^- Non-isotope	233	946	294-2307	3.26	0.236	1.3E-4	0.06	[4]
NO_3^--Total isotope	67	223	109-978	3.33	0.482	4.8E-5	0.21	[11]
NO_3^--Denit Isotope	47	25	9.3-60.6	2.53	0.604	2.1E-4	0.24	[11]
NH_4^+	280	2084	920-4851	3.43	0.069	0.052	0.01	[4]
DOC	98	1198	594-2554	3.19	0.083	0.134	0.01	[25]
FPOM	18	7569	3011-17694	3.62	-0.19	0.062	0.13	[26,27]

a. Preexisting data compiled in [24]
b. New data collected in [24]

Supplementary Figures

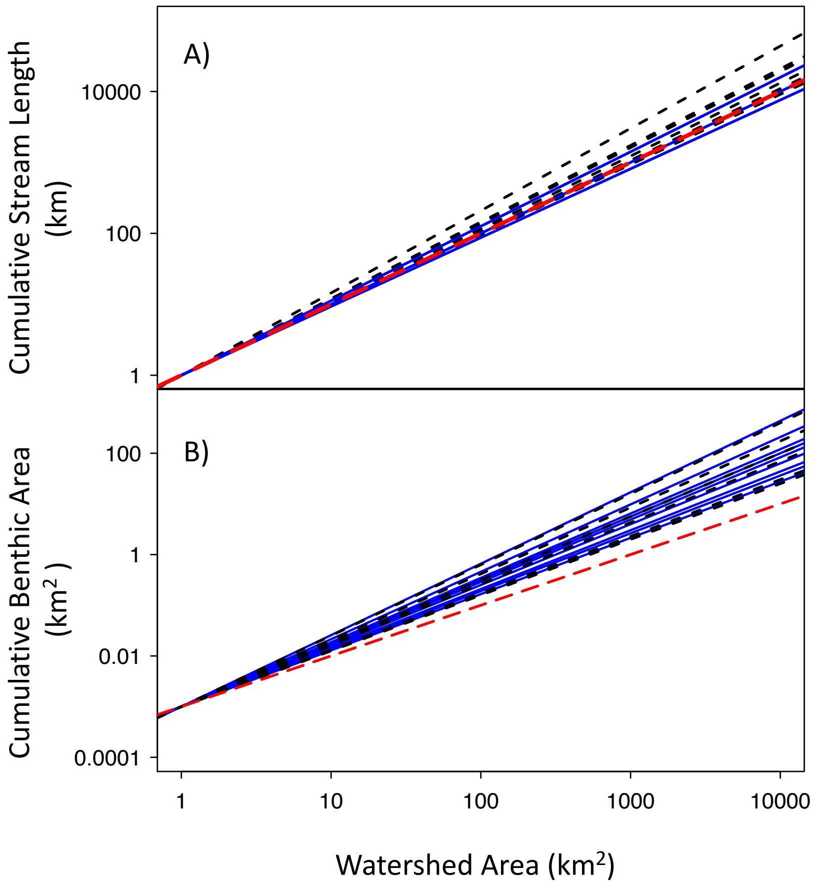

Supplementary Figure 1. Cumulative upstream river length (A), and surface area (B) vs. watershed area. Red dashed lines depict slope = 1. A) dotted black lines depict observed networks from a variety of watersheds in North America (Supplementary Table 2) and solid blue lines represent three types of theoretical channel networks (square, rectangular, narrow; Supplementary Table 1 [28]). B) dotted black lines depict the observed networks with measured *f* [1] and solid blue lines represent the three theoretical channel networks using the observed range of *f* reported in the literature (Supplementary Table 1). The constants for cumulative length and benthic surface area power relationships are normalized to identical values in headwater streams (A = 1 km^2) to emphasize comparison of changes with watershed size.

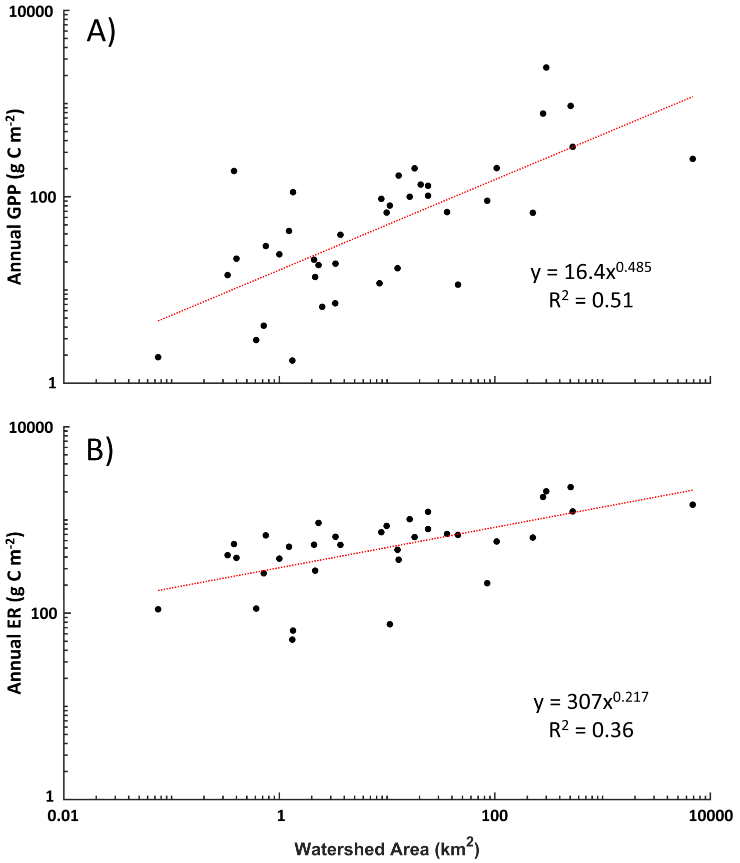

Supplementary Figure 2. Empirical estimates of annual riverine A) GPP and B) ER per unit surface area per year vs. watershed area at the measurement location, synthesized in [10]. Only “unimpacted” streams are included. Annual GPP increases faster than ER with increasing stream size, but all streams are net heterotrophic.

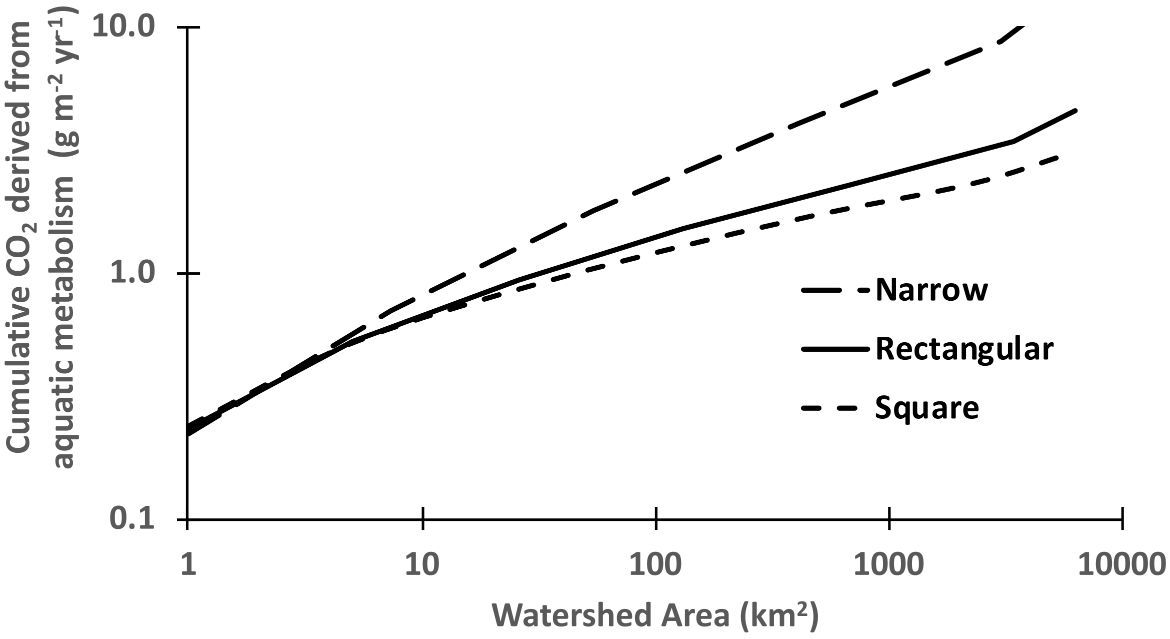

Supplementary Figure 3. Cumulative CO_2 derived from riverine metabolism (cumulative ER - cumulative GPP, normalized to watershed area) in narrow, rectangular, and square river networks at mean annual flow (500 mm yr^{-1}). The relationships of local areal GPP and ER vs. watershed size (*m.local*) applied in these scenarios are shown in Supplementary Figure 2. Each line represents the median from nine model scenarios that reflect potential variation in hydraulic dimensions (Supplementary Table 1).

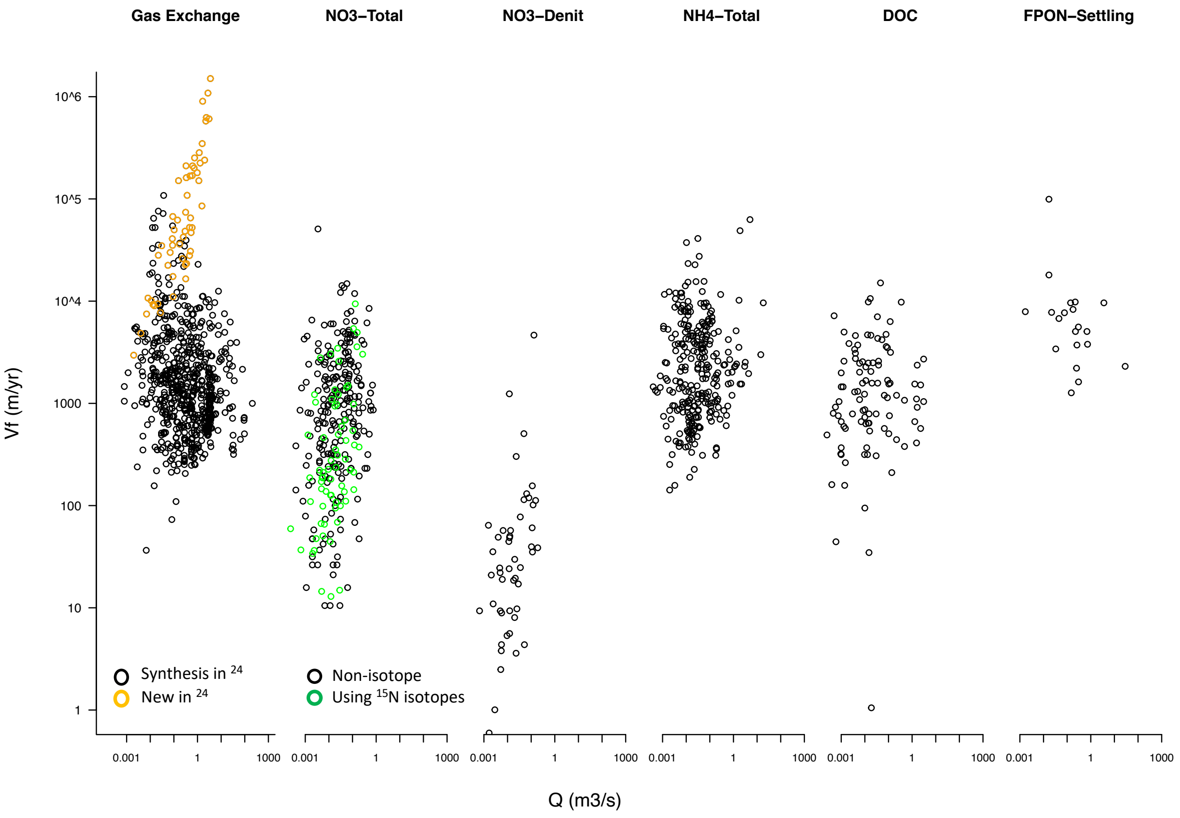

Supplementary Figure 4. Synthesis of local uptake velocities for different constituents in rivers, as a function of discharge at the time of measurement, a rough surrogate for watershed size. See Supplementary Table 5 for literature sources of the data and statistical relationships.

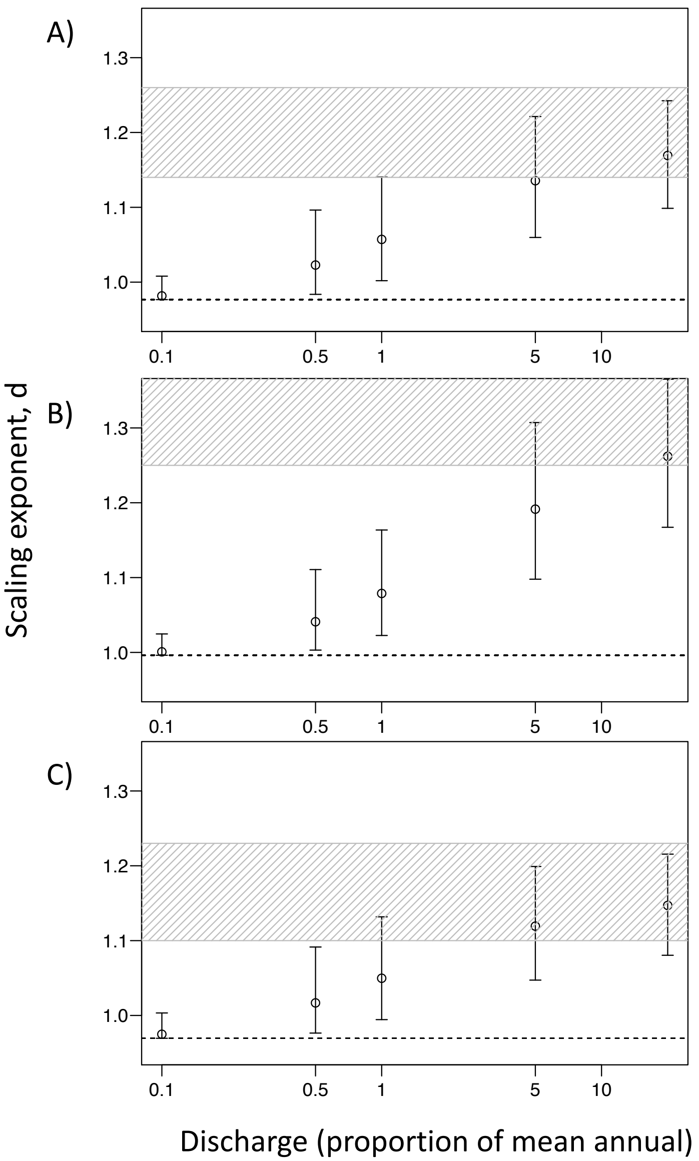

Supplementary Figure 5. Allometric scaling exponent, *d*, of cumulative network-scale biogeochemical function vs. watershed discharge for A) rectangular, B) narrow and C) square river networks. Dashed line shows slope of cumulative river network inputs from the landscape (based on length) versus watershed area, and the shaded area indicates range in slope of cumulative surface area vs. watershed area given variation in hydraulic parameters (varying *e*, *f*, *g*, Supplementary Table 1). Each point shows mean and range from 27 hydraulic scenarios for each network shape, assuming a constant $\upsilon_f = 500$ m yr^{-1}, which is typical of nitrate uptake.

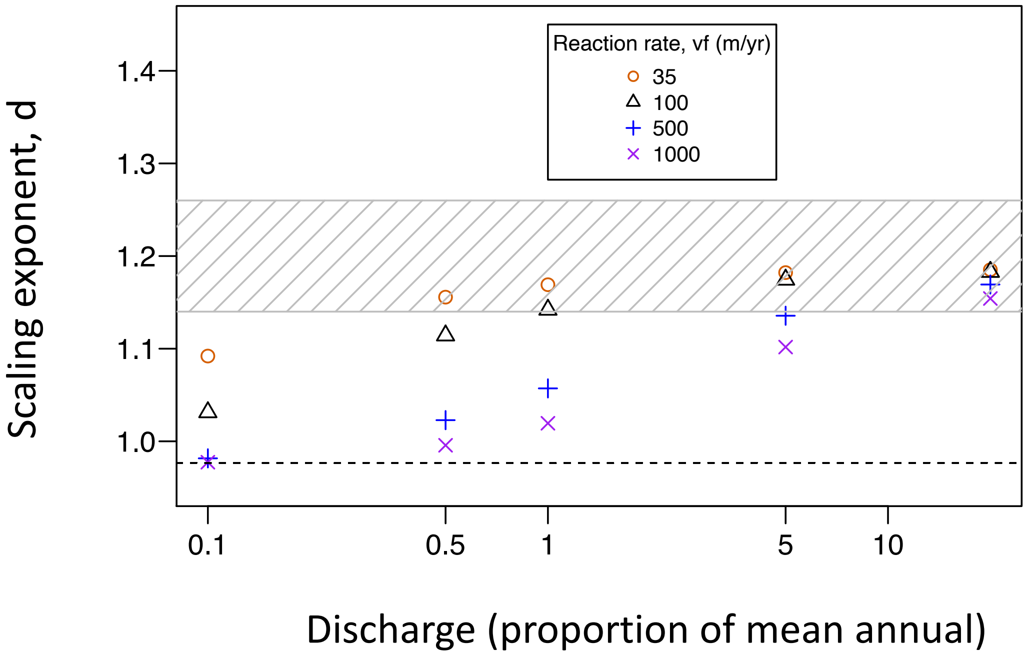

Supplementary Figure 6. Allometric scaling exponent, *d*, of cumulative network-scale biogeochemical function vs. discharge for a rectangular river network for different reaction rates (υ_f) representative of a range of nutrient forms and processes. Dashed line shows slope of cumulative river network inputs from the landscape (based on length) versus watershed area for the rectangular network, and the shaded area indicates range in slope of cumulative surface area vs. watershed area given potential variation in hydraulic parameters (Supplementary Table 1). Each point shows the mean from all scenarios for the rectangular river network, assuming constant υ_f throughout the network (*m.local* = 0).
